# Prevalence and relationship of malnutrition and distress in patients with Cancer using questionnaires

**DOI:** 10.1186/s12885-018-5176-x

**Published:** 2018-12-19

**Authors:** Chenjing Zhu, Baoqing Wang, Yuan Gao, Xuelei Ma

**Affiliations:** 1 Cancer Center, State Key Laboratory of Biotherapy, West China Hospital, Sichuan University, Chengdu, 610041 People’s Republic of China; 2 Department of Oncology, The Second Affiliated Hospital of Xuzhou Medical University, Jiangsu, People’s Republic of China; 3 West China School of Medicine, West China Hospital, Sichuan University, Chengdu, 610041 People’s Republic of China; 4 West China Hospital, Chengdu, 610041 People’s Republic of China

**Keywords:** Cancer, Malnutrition, Psychological

## Abstract

**Background:**

Negative feelings, such as anxiety and depression, are common in patients with cancer. Our aim was to investigate the prevalence of malnutrition and distress in cancer patients and to examine the relationship between them.

**Materials and methods:**

We did a cross-sectional study in West China hospital, China, using adapted questionnaires derived from Patient-Generated Subjective Global Assessment (PG-SGA), Nutritional Risk Screening 2002 (NRS2002) and Distress Thermometer (DT). We also focused on the factors associated with distress.

**Results:**

We found that psychological distress in cancer patients was common, with 39.5% patients suffering from distress. The mean score of PG-SGA was 3.37 (0–6), and 39.1% patients had malnutrition when using 4 as a cut-off value. Meanwhile, the mean score of NRS2002 was 1.91 (0–11), and 25.8% patients presented with malnutrition when using 3 as the cut-off value. Higher scores of nutritional risks confirmed by PG-SGA (*r* = 0.148, *p* < 0.001) and NRS2002 (*r* = 0.142, *p* < 0.001) were significantly correlated with higher levels of psychological stress.

**Conclusion:**

Malnutrition was correlated with psychological stress in cancer patients. Early intervention in the mental problems and nutrition was meaningful, which could improve the psychological statuses of cancer patients.

**Electronic supplementary material:**

The online version of this article (10.1186/s12885-018-5176-x) contains supplementary material, which is available to authorized users.

## Background

Negative feelings, such as anxiety and depression, are common in patients with cancer, which may come from economic status, pain, and treatment-related side effects [1, 2]. Medical and psychological interventions of negative feelings could be of benefit to deal with clinical symptoms such as nausea, vomiting and alopecia [3, 4]. These treatments can relieve the suffering of patients and their families, and may help them receive further life-saving treatments. Therefore, early detection of distress and depression may result in better therapeutic effects [5]. The National Comprehensive Cancer Network (NCCN) guidelines recommend routine screening for psychological distress in cancer patients in all stages using the Distress Thermometer (DT) [6, 7]. A meta-analysis also suggested that DT had a fine diagnostic performance of psychological problems [8].

Malnutrition, due to tumor-related and poor lifestyle-linked factors, is a common, underestimated and multifactorial condition which can occur at any stages of the disease [9, 10]. Regardless of cancer type, the overall prevalence of malnutrition in cancer is about 40%, and has remained constant for the last 30 years [10]. In patients with cancer, severe weight loss prior to treatment varies from 7 to 57%, which may result in increased rates of complications [11–15]. Nutrition problems can be due to mental health issues as well, so the symptoms of malnutrition and psychological distress overlap, but few studies have focused on the relationship between them [16]. Whether malnutrition is an important risk factor of distress is still not clear.

We conducted this study to investigate the prevalence of malnutrition and distress in cancer patients and to examine the relationship between them. We also focused on the factors associated with distress in patients with cancer.

## Methods

### Patients

We conducted a cross-sectional study in West China Hospital, China, which we intended to find out the number of cancer patients with malnutrition and/or distress and the relationship between psychological and nutritional status. From March 2015 to May 2017, we investigated 525 patients with cancer who underwent treatment in West China Hospital. The inclusion criteria were as follows:patients 1) had pathologically confirmed cancer; 2) had no cognitive impairments to understand and fill out a questionnaire by themselves. The exclusion criterion was that patients had a history of mental illness, such as schizophrenia, anxiety disorder, mood disorder, bipolar disorder, and psychoactive substance abuse. Questionnaires assessing depression, anxiety and nutritional status were given to the patients. This study was approved by the Medical Ethics Committee of West China Hospital. Informed consents were signed by the patients if they agreed to participate in the study.

In this survey, we distributed a total number of 525 questionnaires, and the returning rate was 94.5% (496/525). Thirty questionnaires were excluded due to incomplete answers. Finally, 466 questionnaires were valid for the data analyses.

### Questionnaire

We created a questionnaire (see Additional file 1) derived from DT, PG-SGA and NRS2002. In addition to basic demographic information, the questionnaire consisted of Patient-Generated Subjective Global Assessment (PG-SGA) and Nutritional Risk Screening 2002 (NRS2002), both measured the nutritional status of patients. Distress Thermometer (DT) measured the level of patients’ psychological distress.

Demographic information included questions about gender, height, weight, age, residence, marital status, living alone or not, level of education, medical insurance, occupation and religion. The occupation was categorized into having medical background or no medical background, as patients with medical background, for example health care providers, might have a different understanding of their diseases and react differently from other people.

Distress Thermometer is a subjective test to measure the level of distress, which is regarded as the most commonly used tool in detecting distress in cancer patients recently [8]. It consists of a scale from 0 to 10, with 0 indicating no distress and 10 indicating extreme distress. Potential advantages of the DT over other screening tools are its simplicity and acceptability for both health care providers and patients. The cut-off value of the DT is usually 4 recommended by NCCN [6, 7, 17].

Modified from the Subjective Global Assessment, PG-SGA is a brief scale for judging the nutritional statuses of cancer patients which is recommended by the Oncology Nutrition Dietetic Practice Group of the Academy of Nutrition and Dietetics [18, 19]. It brings loss of weight, disease, metabolic stress and physical examination into consideration [20]. Scores range from 0 to 16, with scores of ≥4 suggesting requirements of nutritional support and ≥ 9 indicating a critical demand for nutritional intervention [21].

Recommended by the European Society of Parenteral Enteral Nutrition (ESPEN), NRS2002 is a nutritional risk screening tool in hospitalized patients [22]. The total modules of NRS2002 include three parts: the severity of disease, nutritional status, and age. The cut-off value of NRS2002 is 3, the more severe malnutrition or risk of malnutrition is, the higher the scores will be [23].

### Statistical analysis

We used the SPSS software (version 19.0 Chicago, IL, USA) to analyze the data. We examined the normal distribution of the scores of every patient. Correlation analyses were used to examine the relationship between the nutritional status and psychological distress. Consistency of PG-SGA and NRS2002 was also analyzed. The cut-off value of DT, PG-SGA and NRS2002 were 4, 4 and 3 respectively. Chi-square tests and Fisher’s exact probabilities were performed to examine the influence factors of DT, PG-SGA and NRS2002 scores. A *p* value of less than 0.05 was considered as statistically significant.

## Results

### Demographic analysis

The study population for the current analyses was comprised of 466 individuals, including 257 men and 209 women. The mean age was 50.6 years (SD = 11.9; range 13–81). One hundred and seventy-five participants (37.6%) had a history of breast cancer, 60 patients (12.9%) were diagnosed with malignant lymphoma, 85 (18.2%) had head and neck cancer, other patients with malignant tumors are shown in Table 1. Most of them (*n* = 386, 82.8%) reported having no severe physical pain in our survey. No obvious significant difference was found between men and women.

**Table 1:** Characteristics of the study cohort

Characteristics	N	Constituent ratio (%)
Patients	466	
Age	50.6 ± 11.9 years, 13 to 81 years	
Sex		
Men	209	44.8
Women	257	55.2
Height (cm)		
Men	Mean (SD): 168.2 (5.5)	
Women	Mean (SD): 157.6 (5.4)	
Weight (kg)		
Men Women	Mean (SD): 62.5 (9.9) Mean (SD): 55.4 (8.3)	
BMI		
Men	Mean (SD): 22.1 (3.1)	
Women	Mean (SD): 22.3 (3.0)	
Cancer types		
Head and neck	85	18.2
Breast	175	37.6
Lymphoma	60	12.9
Lung	69	14.8
Gastrointestinal–colorectal	52	11.2
Genitalia–urinary–reproductive	8	1.7
Bone and soft tissue	5	1.1
CNS	6	1.3
Middle ear	1	0.2
Thymus	3	0.6
Unknown/missing	2	0.4
Disease stage		
Early	136	29.2
Intermediate	218	46.8
Advanced	109	23.4
Unknown/missing	3	0.6
Marital status		
Single	21	4.5
Married	428	91.8
Divorced/ Separated	7	1.5
Widowed	10	2.2
Pain		
< 4	386	82.8
≥ 4	80	17.2
Residence		
Country	154	33.0
Suburb	134	28.8
City	178	38.2
Occupation		
Medical background	30	6.9
No medical background	436	93.6
Education background		
Bachelor or above	14	3.0
Undergraduate	56	12.0
Middle-school	286	61.4
Primary school or below	110	23.6
Religious belief		
No	413	88.6
Yes	53	11.4
Source of medical costs		
Medical insurance	261	56.0
New Rural Cooperative Medical System	137	29.4
Self-supporting	51	10.9
Publicly-funded	11	2.4
Other	6	1.3

### Prevalence of distress and malnutrition

We used the cut-off score of 4 recommended by the NCCN [7] to identify the level of distress. In this study, 184 patients (39.5%) suffered from distress. The mean distress score was 3.7 with a standard deviation of 2.7, ranging from 0 to 10.

Nutritional risk was identified by PG-SGA and NRS2002. The mean score of PG-SGA was 3.37 (ranging from 0 to 6), and 39.1% patients (182/466) showed positive results when using 4 as a cut-off value. Meanwhile, the mean score of NRS2002 was 1.91 (0–11), and 120 patients (25.8%) suffered from malnutrition when using 3 as the cut-off value.

In addition, we performed serial and parallel tests respectively. Serial test was defined by the scores of PG-SGA ≥ 4 and NRS 2002 ≥ 3 simultaneously as malnutrition. As a result, 85 patients (18.2%) were in the malnutrition status. Parallel test was defined by either the score of PG-SGA ≥ 4 or the score of NRS 2002 ≥ 3 as positive results, and the prevalence was 46.6% (217/466) (see Table 3).

### Relationship between nutrition and distress

In general, higher scores of nutritional risks confirmed by PG-SGA (*r* = 0.148, *p* < 0.001) and NRS2002 (*r* = 0.142, p < 0.001) were positively correlated with higher levels of psychological stress (see Table 2). The same result was demonstrated by a series of chi-square tests using the standard of PG-SGA ≥ 4, NRS 2002 ≥ 3 and DT ≥ 4. Table 3 presents the results of the relationship between PG-SGA, NRS2002, PG-SGA plus NRS2002, PG-SGA or NRS2002 and DT scores, respectively. PG-SGA (score = 6.512, *p* = 0.011 < 0.05) and NRS2002 (score = 6.340, *p* = 0.012 < 0.05) showed positive correlation with DT. The strongest correlation appeared in parallel test (score = 9.610, *p* = 0.002 < 0.05), but there was no statistical significance in serial test (score = 4.287, *p* = 0.38 > 0.05).

**Table 2:** Correlation analyses demonstrating the relationship between Distress Thermometer scores and other factors

	Correlations with Distress Thermometer
PG-SGA scores	r = 0.148, *p* < 0.001
NRS2002 scores	r = 0.142, *p* < 0.001

*PG-SGA*: Patient-Generated Subjective Global Assessment

*NRS2002*: Nutritional Risk Screening 2002

**Table 3:** Chi-square analyses demonstrating elevated Distress Thermometer scores in patients with poor nutritional status

Nutritional status	Distress		N
PG-SGA score	No	Yes	
< 4	185	99	284
≥ 4	97	85	182
Overall	282	184	466
	6.512, *p* = 0.011 < 0.05		
NRS2002 score			
< 3	221	125	346
≥ 3	61	59	120
Overall	282	184	466
	6.340, *p* = 0.012 < 0.05		
Serial test: PG-SGA and NRS2002			
No	239	142	381
Yes	43	42	85
Overall	282	184	466
	4.287, *p* = 0.38>0.05		
Parallel test: PG-SGA or NRS2002			
No	167	82	249
Yes	115	102	217
Overall	282	184	466
	9.610, *p* = 0.002 < 0.05		

*PG-SGA*: Patient-Generated Subjective Global Assessment

*NRS2002*: Nutritional Risk Screening 2002

Serial test was defined by the scores of PG-SGA ≥ 4 and NRS 2002 ≥ 3 simultaneously

Parallel test was defined by either the score of PG-SGA ≥ 4 or NRS 2002 ≥ 3

### Other factors associated with distress

To examine whether socio-demographic characteristics have effects on the level of distress, a series of analyses (Chi-square or correlation tests) were conducted. The factors affecting DT scores of patients were old age (*p* < 0.01) and pain (*p* < 0.01). The association was not statistically significant between DT scores and other socio-demographic and clinical variables, including age, gender, marital status, disease stage or education.

### Other factors associated with nutrition

We also conducted Chi-square or correlation tests to verify whether socio-demographic characteristics have impacts on the level of nutritional status. With regard to nutrition, no significant relationship was found between PG-SGA scores and any demographic and clinical variables except for gender (*p* < 0.01) and disease stage (*p* < 0.05). Meanwhile, NRS2002 scores were significantly correlated with age (*p* < 0.05), gender (*p* < 0.01), disease stage (*p* < 0.01), pain (*p* < 0.01), and occupation (*p* < 0.05) (see Table 4).

**Table 4:** Chi-square analyses demonstrating the relationship between different basic situations and DT, PG-SGA and NRS2002

Basic situations	Relationship with		
	DT	PG-SGA	NRS2002
Age (> 55)	7.354^b^	1.807	5.174^a^
Gender	2.638	11.002^b^	11.879^b^
Marital status	4.433	6.426	1.80
Disease stage	6.135	7.196^a^	11.217^b^
Pain	47.456^b^	2.100	6.973^b^
Residence	2.831	2.698	1.530
Occupation	2.704	1.879	6.813^a^
Education background	1.801	1.517	2.269
Religious belief	0.841	1.975	1.482
Source of medical costs	8.700	5.240	4.715

a: *p* < 0.05

b: *p* < 0.01

*PG-SGA*: Patient-Generated Subjective Global Assessment

*NRS2002*: Nutritional Risk Screening 2002

*DT*: Distress Thermometer

## Discussion

In this cross-sectional study, we found that psychological distress in cancer patients was common (39.5%). Positive results which arose from correlation analyses suggested that the nutritional status might have a positive relationship with psychological level (*p* < 0.001). The factors associated with distress in cancer patients were old age and pain. Age, gender, disease stage, pain, and occupation might affect their nutritional status.

The prevalence of distress was 39.5% in our survey. Previous studies which used other psychological assessment scales reported similar results, for example, the percentage of cancer patients with psychological distress measured by the Brief Symptom Inventory-18 was reported as 37.8% [24], and by Self-Rating-Depression-Scale (SDS) the prevalence was 34.3% [25]. Detection rate of distress in cancer patients ranged from 3 to 31% measured by Hospital Anxiety and Depression Scale (HADS) [25].

The mildly positive associations between DT scores and PG-SGA scores (*r* = 0.148, *p* < 0.001) and between DT scores and NRS2002 scores (*r* = 0.142, *p* < 0.001) implicated that malnutrition was independently correlated to psychological distress. Several previous studies have reported similar results in breast, lung and head and neck cancer patients [16, 26, 27], especially in patients who were treated with radiation therapy [16]. However, causality between these two factors is not clear yet. Previous studies have reported that pro-inflammatory cytokines, including IL-1, IL-6, TNF-α, and INF-γ [28–31], were associated with depressive symptoms in cancer patients. Depression, fatigue and cognitive impairment, were the systemic effects of these cytokines, which might influence the quality of life of patients before, during and after treatment [29]. Psychological factors such as patient’s acceptance of the severity of disease and any concomitant change in behavior might also contribute to the link between these two syndromes [16]. Further studies are needed to clarify these problems.

Moreover, correlation analyses and chi-square tests (see Table 4) showed that age and pain were the influence factors of DT scores. Age, gender, disease stage, pain and occupation could influence NRS2002 and PG-SGA scores. Several studies have reported that both pain and age have independent correlations with DT scores [1, 3, 4], which was in accordance with our study. Besides PG-SGA and NRS2002, mini nutritional assessment and malnutrition screening tools are also widely accepted nutritional scales in clinical practice. Previous researches using these scales found that nutritional status in patients with cancer could be influenced by age, gender and pain [32–34], but few of them mentioned the effect of occupation. In our study, we considered that occupation was related to economic conditions and working duration of the patients, thus might influence their diet structures which play a role in their nutritional status.

There were several limitations in our study. Firstly, this study was a cross-sectional study, which could only provide clues for a causality relationship between these two factors. Whether malnutrition caused distress or distress led to malnutrition was unknown and had to be further confirmed by a follow-up study. Secondly, our study was a single-center research and most participants were with heavily pre-treated diseases, whereas a multi-center study would be more supportive of our results. Thirdly, this study was a questionnaire-based survey, and interview-based surveys would be more reliable though time-consuming and energy-consuming. Finally, our study included various types of cancer and we didn’t analyze every one of them. To further elucidate the relationship of malnutrition and distress, future researches are still in demand.

## Conclusions

Detecting distress in patients early was possible with various scales. The results of our study might help to provide new ideas of diagnosis and treatment of distress. As a large proportion of patients with cancer had a DT score of 4 or above, it was meaningful for health care providers to intervene in their psychological problems. Social workers may provide counselling about certain psychosocial problems, and psychologists may offer help in addressing more serious problems. What’s more, malnutrition was closely related to distress in cancer patients, indicating that food-based interventions were needed to improve psychological status of cancer patients. Our manuscript highlights the need of an interdisciplinary collaboration plan made by physicians, nurses, dietitians, social workers, psychologists etc. to maintain an acceptable quality of life and improve clinical outcomes of patients.

## Additional file

Additional file 1: Questionnaires derived from DT, PG-SGA and NRS2002 which contain questions about basic demographic information, nutritional status, and level of psychological distress of patients. (DOCX 653 kb)

## Abbreviations

DT: Distress Thermometer
NRS2002: Nutritional Risk Screening 2002
PG-SGA: Patient-Generated Subjective Global Assessment

## References

[1] Jadoon NA (2010). Assessment of depression and anxiety in adult cancer outpatients: a cross-sectional study. BMC Cancer.

[2] Pirl WF (2004). Evidence report on the occurrence, assessment, and treatment of depression in cancer patients. J Natl Cancer Inst Monogr.

[3] Park EM (2016). Parenting concerns, quality of life, and psychological distress in patients with advanced cancer. Psychooncology.

[4] Park S (2016). Risk factors for postoperative anxiety and depression after surgical treatment for lung cancerdagger. Eur J Cardiothorac Surg.

[5] Lloyd-Williams M (2000). Difficulties in diagnosing and treating depression in the terminally ill cancer patient. Postgrad Med J.

[6] National Comprehensive Cancer (2003). N., *Distress management. Clinical practice guidelines*. J Natl Compr Cancer Netw.

[7] Holland JC, Bultz BD, N. National Comprehensive Cancer (2007). *The NCCN guideline for distress management: a case for making distress the sixth vital sign*. J Natl Compr Cancer Netw.

[8] Ma X (2014). The diagnostic role of a short screening tool--the distress thermometer: a meta-analysis. Support Care Cancer.

[9] Capra S, Ferguson M, Ried K (2001). Cancer: impact of nutrition intervention outcome--nutrition issues for patients. Nutrition.

[10] Righini CA (2013). Assessment of nutritional status at the time of diagnosis in patients treated for head and neck cancer. Eur Ann Otorhinolaryngol Head Neck Dis.

[11] Datema FR, Ferrier MB, Baatenburg de Jong RJ (2011). *Impact of severe malnutrition on short-term mortality and overall survival in head and neck cancer*. Oral Oncol.

[12] Ghadjar P (2015). Impact of weight loss on survival after chemoradiation for locally advanced head and neck cancer: secondary results of a randomized phase III trial (SAKK 10/94). Radiat Oncol.

[13] Jager-Wittenaar H (2011). Changes in nutritional status and dietary intake during and after head and neck cancer treatment. Head Neck.

[14] Jager-Wittenaar H (2007). Critical weight loss in head and neck cancer--prevalence and risk factors at diagnosis: an explorative study. Support Care Cancer.

[15] Langius JA (2016). Prediction model to predict critical weight loss in patients with head and neck cancer during (chemo)radiotherapy. Oral Oncol.

[16] Ma L (2013). The association between malnutrition and psychological distress in patients with advanced head-and-neck cancer. Curr Oncol.

[17] Hong JS, Tian J (2013). Sensitivity and specificity of the distress thermometer in screening for distress in long-term nasopharyngeal cancer survivors. Curr Oncol.

[18] Bauer J, Capra S, Ferguson M (2002). Use of the scored patient-generated subjective global assessment (PG-SGA) as a nutrition assessment tool in patients with cancer. Eur J Clin Nutr.

[19] Rodrigues CS, Chaves GV (2015). Patient-generated subjective global assessment in relation to site, stage of the illness, reason for hospital admission, and mortality in patients with gynecological tumors. Support Care Cancer.

[20] Shaw C (2015). Comparison of a novel, simple nutrition screening tool for adult oncology inpatients and the malnutrition screening tool (MST) against the patient-generated subjective global assessment (PG-SGA). Support Care Cancer.

[21] Isenring E, Bauer J, Capra S (2003). The scored patient-generated subjective global assessment (PG-SGA) and its association with quality of life in ambulatory patients receiving radiotherapy. Eur J Clin Nutr.

[22] Kondrup J (2003). Nutritional risk screening (NRS 2002): a new method based on an analysis of controlled clinical trials. Clin Nutr.

[23] Kondrup J (2003). ESPEN guidelines for nutrition screening 2002. Clin Nutr.

[24] Carlson LE (2004). High levels of untreated distress and fatigue in cancer patients. Br J Cancer.

[25] Neilson KA (2010). Psychological distress (depression and anxiety) in people with head and neck cancers. Med J Aust.

[26] Saxton JM (2014). Effects of an exercise and hypocaloric healthy eating intervention on indices of psychological health status, hypothalamic-pituitary-adrenal axis regulation and immune function after early-stage breast cancer: a randomised controlled trial. Breast Cancer Res.

[27] Chabowski M (2018). Is nutritional status associated with the level of anxiety, depression and pain in patients with lung cancer?. J Thorac Dis.

[28] Musselman DL (2001). Higher than normal plasma interleukin-6 concentrations in cancer patients with depression: preliminary findings. Am J Psychiatry.

[29] Seruga B (2008). Cytokines and their relationship to the symptoms and outcome of cancer. Nat Rev Cancer.

[30] Tisdale MJ (2009). Mechanisms of cancer cachexia. Physiol Rev.

[31] Inagaki M (2013). Associations of interleukin-6 with vegetative but not affective depressive symptoms in terminally ill cancer patients. Support Care Cancer.

[32] Orell-Kotikangas H (2015). NRS-2002 for pre-treatment nutritional risk screening and nutritional status assessment in head and neck cancer patients. Support Care Cancer.

[33] Araujo dos Santos C (2015). Patient-generated subjective global assessment and classic anthropometry: comparison between the methods in detection of malnutrition among elderly with cancer. Nutr Hosp.

[34] Bossola M (2015). Nutritional interventions in head and neck cancer patients undergoing chemoradiotherapy: a narrative review. Nutrients.

