# Supplementary material for: Prevalence and relationship of malnutrition and distress in patients with Cancer using questionnaires
Source: BMC Cancer. 2018 Dec 19;18:1272. doi: 10.1186/s12885-018-5176-x (PMC6299972; doi:10.1186/s12885-018-5176-x)
Supplement: Supplementary file 1 — Questionnaires derived from DT, PG-SGA and NRS2002 which contain questions about basic demographic information, nutritional status, and level of psychological distress of patients. (DOCX 653 kb) [file 12885_2018_5176_MOESM1_ESM.docx]

Bed number Patient ID

Telephone number Date

**I. General information**

1. Your age: Sex: M □ F □

Height: cm Weight: kg

2. Your marital status：

A. single B. married C. divorced/separated D. widowed

3. Your living status：

A. living alone B. living with family C. other

4. where you live:

A. country B. suburb C. city

5. Your occupation before admission into the hospital:

A. medical related (please choose: doctor □ nurse □ Pharmaceutical related □ other )

B. non-medical related (please choose: worker □ farmer □ teacher □ Civil servant □ Self-employed person □ other )

6. Your education level：

A bachelor degree or above B. undergraduate C. middle school D. primary school or below

7. Your religious belief

A. no

B. yes (please choose: Buddhism □ Taoism □ Christianity □ Catholicism □ Islamism □ other )

1. The source of your medical expenses:

A. Medical insurance B. New rural cooperative medical system C. self-supporting D. publicly-funded E. other

9 The disease you are suffering from is

10 The stage of your disease：

A. early B. intermediate C. advanced D. do not know

11. Do you have any relatives who can take care of you and accompany you recently?

A. no

B. yes (please choose: wife/husband □ son/daughter □ parents □ brothers/sisters □ other )

12. Do you suffer from any other diseases?

A．no

B. yes (please choose: hypertension □ diabetes □ Heart diseases □ cirrhosis □ other_______)

13. Have the doctors discussed the nutrition plan with you?

A. not yet

B. yes (please choose with whom to discuss: me □ my family members □ me and my family members □）

14. Do you think your nutrition at this stage needs to be strengthened?

A. very much B. a little C.no

15. Are you worried about your future nutritional status?

A. very much B. a little C. never thought about it D. not worried at all

16. Have you received medical nutrition support?

A. yes (please choose: Enteral nutrition □ Parenteral nutrition □ both □） B.no

17. Are you willing to accept medical nutrition support?

A. yes B. willing to try if necessary C.no (please choose why: effect is not obvious □ too expensive □ taste is too bad □ worried about being infected with other diseases □ other_______)

18. How do you supplement your nutrition now?

A. dietetic invigoration B. enteral nutrition C. parenteral nutrition (fluid infusion) D. did not do anything

19. Have you ever received radiotherapy? (If not, please jump to the twenty-fifth question)

A. No B. Yes (please choose how many courses you have experienced: 1 □ 2 □ 3 □ more than 3 □)

20. What complications have you had after radiotherapy?

Dry mouth □ problems swallowing □ Mucocutaneous reaction □ nausea/vomiting □ Dizziness/headache □ fatigue/weakness □ other

21. Do the complications affect your life?

A. very much B. a relatively large impact on normal life C. a modest impact D. no effect

22. Does radiotherapy affect your nutritional status?

A. very much B. a relatively large impact on normal life C. a modest impact D. no effect

23. Are you satisfied with the efficacy of radiotherapy?

A. quite B. relatively C. a little D. not very satisfied E. very dissatisfied F. do not know

24. Your biggest concern about radiotherapy is

25. Please assess your current pain level (0 as painless, 1-3 as mild, 4-6 as moderate, and 7-10 as severe pain. Circle a number that best represents the degree of pain)


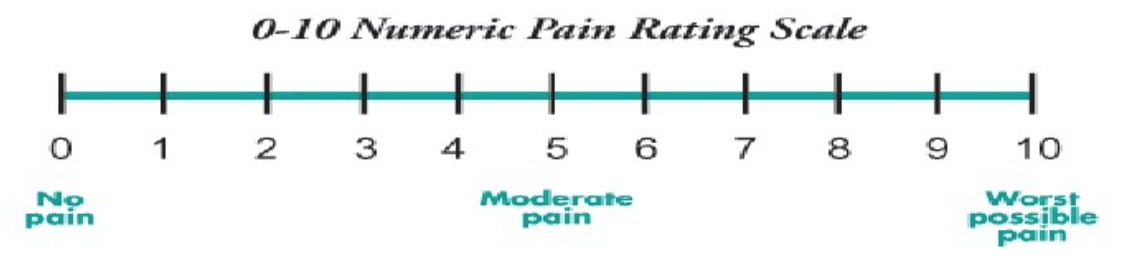


II. Psychological Distress Thermometer and related problems

Instructions:

First please circle the number (0-10) that best describes how much distress you have been experiencing in the past week including today.

Extreme distress

No distress


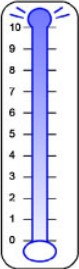


Second, please indicate if any of the following has been a problem for you in the past week including today. Be sure to check YES or NO for each.


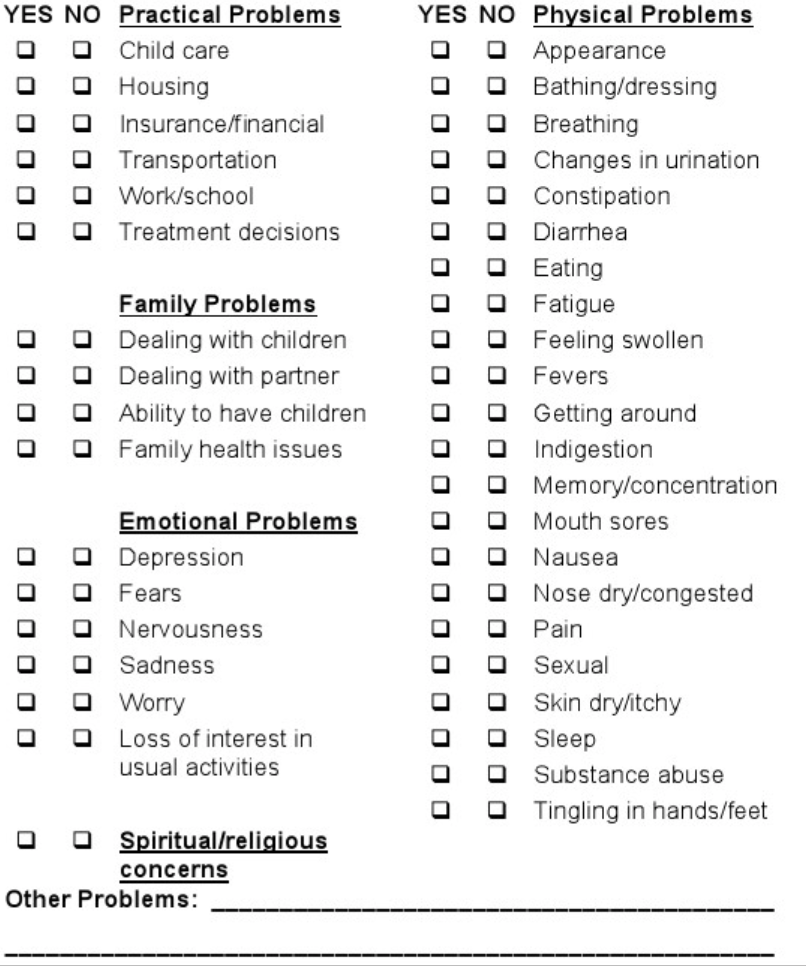


III. Nutritional risk screening assessment form

1. Disease status

If you have the following conditions, please tick in the box.

Tumor □ Cirrhosis □ COPD □ Long term hemodialysis □ Diabetes □ Stroke □ Major abdominal surgery □ Severe pneumonia □ Hematological malignancies □ Craniocerebral injury □ Myelosuppression □ Acute inflammatory reaction □ Pulmonary or cardiac cachexia □ Trauma □ Bedsore, open wound or fistula □

1. Nutritional status

One month ago you weighed about kg

Six months ago you weighed about kg

3. Have you lost weight recently (Wt loss > 5%)？

A. No B. Yes (please choose: in 1 mth □ in 2 mths □ in 3 mths □ in 6 mths □)

4. Have you had a reduced dietary intake in the last week?

A. No B. a 20%~50% reduction C. a 50%~75% reduction D. a 70%~100% reduction

5. Over the past month, your activity level:

A. normal with no limitations B. able to be up and about with fairly normal activities C. not feeling up to most things, but in bed or chair less than half the day C. able to do little activity and spend most of the day in bed or chair D. pretty much bedridden, rarely out of bed

6. Have you had a fever in the last week？

A. No B. Yes（please choose: within 72hrs □ 72hrs□ more than 72hrs□）

7. If you have had a fever, the highest temperature in the past week was ℃

8. Are you currently on steroids or has been on steroids in the past month?

A. No B. <10mg prednisone equivalents/day C. ≥ 10mg and < 30mg prednisone equivalents/day D. ≥ 30mg prednisone equivalents/day

The remainder of the form will be completed by your doctor, nurse, or therapist. Thank you.
